# Keeping It in the Family: the Childhood Burden of Tuberculosis

**DOI:** 10.3201/eid2303.AC2303

**Published:** 2017-03

**Authors:** Terence Chorba, John Jereb

**Affiliations:** Centers for Disease Control and Prevention, Atlanta, Georgia, USA

**Keywords:** art science connection, emerging infectious diseases, art and medicine, about the cover, tuberculosis, tuberculosis and other mycobacteria, Mycobacterium tuberculosis, drug-resistant tuberculosis, infectious diseases, Das Kind und der Tod, The Child and Death, Edvard Munch, keeping it in the family: the childhood burden of tuberculosis, public health

**Figure:**
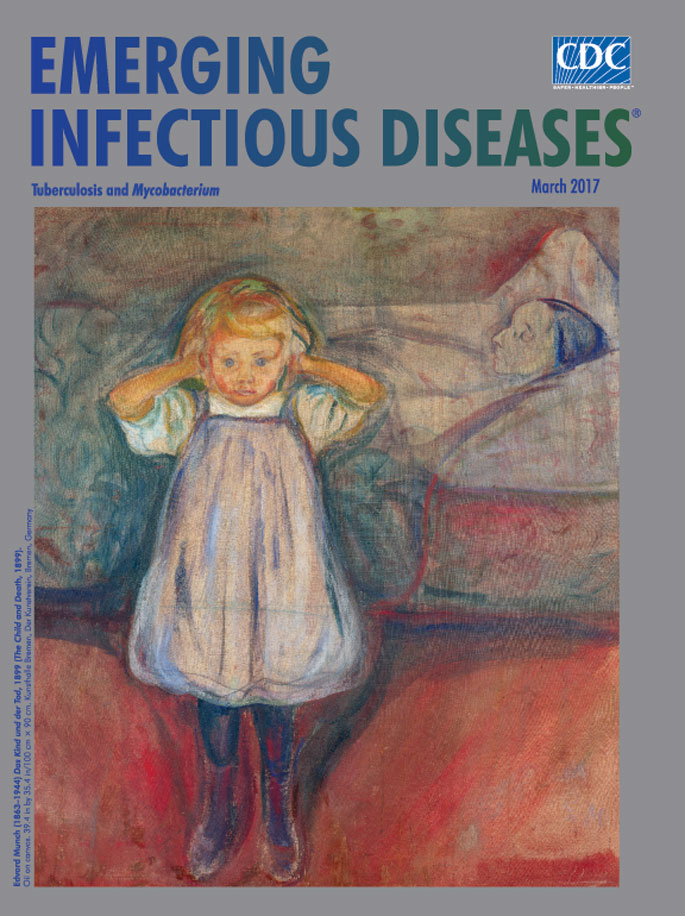
**Edvard Munch (1863−1944) Das Kind und der Tod, 1899 (The Child and Death, 1899). Oil on canvas. 39.4 in by 35.4 in/100 cm × 90 cm.** Public domain image. Art located at Kunsthalle Bremen – Der Kunstverein in Bremen, Germany.

“My art must be seen against the background of the heavy freight of my inheritance,–tuberculosis on Mother’s side, mental illness on Father’s side (Grandfather’s phthisis),–my art is a self confession…” “The illness followed me all through my childhood and youth,–the germ of consumption placed its blood-red banner victoriously on the white handkerchief.”

—Edvard Munch

Edvard Munch, born in December 1863, was the second of 5 children of Laura Bjølstad and Christian Munch, a physician, in Løten, Norway. As an infant, he moved with his family to Christiania (now Oslo). There Laura died in 1868 of tuberculosis (TB), after which Christian dealt with profound depression. At the time of Laura’s death, 14 years before Robert Koch announced that *Mycobacterium tuberculosis* was the cause of the disease, an estimated 285 persons per 100,000 died of phthisis (pulmonary TB or a similar progressive systemic disease) annually in Norway; most deaths occurred among those of child-bearing age. In 1896–1900, after the technique for diagnostic sputum smears was widely known and practiced, the death rate from TB in Norway was 415 per 100,000 for women 20–39 years of age; the difference from the earlier number perhaps reflected increased diagnostic acumen.

In his memoirs, Munch recalled the Christmas when, at age 5, he stood with his 6-year-old sister (Johanne Sophie) and his younger siblings at their mother’s bedside. Sophie sang “Silent Night,” and Laura kissed each child. Shortly thereafter, Laura died. Munch later portrayed the desperation of a child clutching her head at her mother’s death, in *The Child and Death*, featured on this month’s cover.

In *The Child and Death,* Munch captures the innocence of childhood disrupted by terrible circumstances, made more heartbreaking because the mother’s death portends the daughter’s death—the infection has been transmitted already. Munch knew what awaited his sister: her wide-eyed gaze shames the viewer/voyeur who has drawn closer to inquire about warm flesh tones against a background of gray-blue pallor, while bloody carmine smudges the bed and creeps around the girl.

The World Health Organization (WHO) has estimated that 9.7 million children (aged <15 years) are now orphans because of TB. In addition to the social and psychological burden of TB, children themselves account for a considerable portion of the associated morbidity and mortality. WHO estimates that 10.4 million new (incident) TB cases occurred in 2015, of which 5.9 million were in men, 3.5 million in women, and 1.0 million in children. The diagnosis and treatment of childhood TB are often problematic. Adequate sputum samples are difficult to obtain from children, thus hindering timely diagnosis. There is also relative lack of drug formulations for children, despite recent introductions of user-friendly fixed-dose combinations.

Sophie died from TB at age 15, a year after Munch himself took ill with the disease. Munch recalled the pathos of Sophie’s death in the painting *The Sick Child* (1886), featured as EID’s cover art in March 2011. Munch’s account of his own illness is poignant:

“‘Papa the stuff I am spitting is so dark.’

‘Is it, my boy?’

He brought the candle….Next time I spat on the sheet to see what it was.

‘It is blood Papa.’

He stroked my hair – ‘Don’t be afraid, my boy.’

So I had tuberculosis. There was so much talk about it. When you spat blood you had tuberculosis….

‘Don’t be frightened boy,’ Father said again.

‘When you spit blood you have tuberculosis,’ I said and I coughed again and got more blood.”

Munch’s survival was unexpected: in the pre-antimicrobial drug era, the case-fatality rate for TB was 70%. Although Munch also nearly died of influenza in the pandemic of 1918–19, he survived, recovered, and died in 1944, at age 80, at his country home in Ekley, Norway.

Globally the epidemics of drug-resistant TB, multi-drug-resistant TB, and extensively drug-resistant TB are formidable. Almost 10% of *M. tuberculosis* isolates in the United States and 20% of isolates worldwide are resistant to at least one first-line TB drug, mostly to isoniazid. Drug resistance is associated with greater morbidity, accounts for almost 25% of global TB mortality, and requires treatment that is more costly, more difficult, and of greater duration. These circumstances threaten to reverse the antimicrobial gains against TB, pushing us toward a world that may more resemble the pre-antibiotic era in which Edvard Munch’s mother and sister died, and in which he somehow survived to bring us the ghosts of his memories.
